# Supplementary material for: Pathophysiological Significance of Neutrophilic Transfer RNA-Derived Small RNAs in Asymptomatic Moyamoya Disease
Source: Cells. 2021 May 1;10(5):1086. doi: 10.3390/cells10051086 (PMC8147334; doi:10.3390/cells10051086)

2D Structure	Local AU	Position

```
1174                                    1204
                              8mer-1a
5'-ctGCGGGGTTTAGGTTAGATGTCACGGAAAa-3' utr
     :|| |:|     ::||  |: | |||||||
3'-uuUGCACUAUUGGUGAUGUG-A-UGCCUUUg-5' tRF
                16151413        7 6 5 4 3 2
                3' Pairing        Seed
```

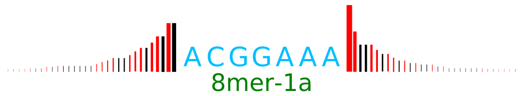

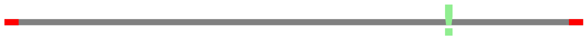

Supplement: Supplementary file 1 [file cells-10-01086-s001.zip › cells-1149161 3rd supplementary materials/Data Supplements/Data Supplement V.zip!/Data supplement 6-mRNAs targets of tsRNAs/tsRNA-Val-AAC-017/Diagrams/tRF-Val-AAC-017_vs_ENST00000290524(RFX5).pdf]
